# Replicative Senescence-Associated LINE1 Methylation and LINE1-Alu Expression Levels in Human Endothelial Cells

**DOI:** 10.3390/cells11233799

**Published:** 2022-11-27

**Authors:** Deborah Ramini, Silvia Latini, Angelica Giuliani, Giulia Matacchione, Jacopo Sabbatinelli, Emanuela Mensà, Maria Giulia Bacalini, Paolo Garagnani, Maria Rita Rippo, Giuseppe Bronte, Massimiliano Bonafè, Maurizio Cardelli, Fabiola Olivieri

**Affiliations:** 1 Clinic of Laboratory and Precision Medicine, IRCCS INRCA, 60121 Ancona, Italy; 2 Department of Clinical and Molecular Sciences, Università Politecnica delle Marche, 60126 Ancona, Italy; 3 Laboratory Medicine Unit, Azienda Ospedaliero Universitaria delle Marche, 60126 Ancona, Italy; 4 IRCCS Istituto delle Scienze Neurologiche di Bologna, 40139 Bologna, Italy; 5 Department of Experimental, Diagnostic and Specialty Medicine (DIMES), University of Bologna, 40126 Bologna, Italy; 6 Applied Biomedical Research Center (CRBA), S. Orsola-Malpighi Polyclinic, 40126 Bologna, Italy; 7 CNR Institute of Molecular Genetics “Luigi Luca Cavalli-Sforza”-Unit of Bologna, 40126 Bologna, Italy; 8 Department of Laboratory Medicine, Clinical Chemistry, Karolinska Institutet, Karolinska University Hospital, 141 86 Huddinge, Sweden; 9 Advanced Technology Center for Aging Research, IRCCS INRCA, 60121 Ancona, Italy

**Keywords:** cellular senescence, Alu sequences, retrotransposable elements, LINE1

## Abstract

One of the main challenges of current research on aging is to identify the complex epigenetic mechanisms involved in the acquisition of the cellular senescent phenotype. Despite some evidence suggested that epigenetic changes of DNA repetitive elements, including transposable elements (TE) sequences, are associated with replicative senescence of fibroblasts, data on different types of cells are scarce. We previously analysed genome-wide DNA methylation of young and replicative senescent human endothelial cells (HUVECs), highlighting increased levels of demethylated sequences in senescent cells. Here, we aligned the most significantly demethylated single CpG sites to the reference genome and annotated their localization inside TE sequences and found a significant hypomethylation of sequences belonging to the Long-Interspersed Element-1 (LINE-1 or L1) subfamilies L1M, L1P, and L1HS. To verify the hypothesis that L1 demethylation could be associated with increased transcription/activation of L1s and/or Alu elements (non-autonomous retroelements that usually depend on L1 sequences for reverse transcription and retrotransposition), we quantified the RNA expression levels of both L1 (generic L1 elements or site-specific L1PA2 on chromosome 14) and Alu elements in young and senescent HUVECs and human dermal fibroblasts (NHDFs). The RNA expression of Alu and L1 sequences was significantly increased in both senescent HUVECs and NHDFs, whereas the RNA transcript of L1PA2 on chromosome 14 was not significantly modulated in senescent cells. Moreover, we found an increased amount of TE DNA copies in the cytoplasm of senescent HUVECs and NHDFs. Our results support the hypothesis that TE, which are significantly increased in senescent cells, could be retrotranscribed to DNA sequences.

## 1. Introduction

Numerous epigenetic alterations occur in each tissue over time and may cause some of the physiological changes associated with normal aging and age-related diseases (ARD) [1,2]. Age-related epigenic changes involve alterations in the DNA methylation pattern, post transcriptional histone modifications, remodelling of chromatin and transcription of different classes of non-coding RNAs [3,4]. Methylation changes occur predominantly in CpG islands, i.e., CG-rich sequences that can be methylated [5]. Different sets of CpG sites whose DNA methylation levels can measure age in humans and other vertebrates were identified [6,7,8,9]. The methylation level observed at single CpG sites, can show either an increase or a decrease during aging with respect to younger ages. During aging, there is a tendency to increase DNA methylation in specific sites which are mainly located in gene-promoter regions [10,11,12]; however, the average of CpG methylation in the genome exhibits a general tendency to decrease in aged cells or tissues, especially in repetitive DNA sequences, belonging to constitutive heterochromatin domains [13,14]. Indeed, global DNA methylation, measured in in vitro cultured human derived cells, i.e., fibroblasts and human endothelial cells, significantly decreases during cellular senescence [15,16,17]. Overall, as the loss of DNA methylation promotes the formation of transcribed silent heterochromatin this change will facilitate the loss of heterochromatin [18]. More in detail, in eukaryotic cells, the phenomenon of cellular senescence is associated with epigenetic alteration of chromatin regions containing transposable elements (TEs) [19]. The TEs (previously defined as ‘jumping genes’) are discrete pieces of DNA that can move within (and sometimes between) genomes. The methylation of repetitive elements, TEs included, is considered largely responsible of the average CpG methylation in the human genome and therefore sometimes it is used as a surrogate marker of global genome methylation [20]. This assumption is justified by the observation that, in the mammalian genome, most of the CpG sites are contained in TEs. Interestingly, the age-related loss of methylation appears weaker in centenarians, suggesting that there are inheritable genetic traits predisposing for longevity and delayed epigenetic changes of the global TEs [21].

Different classes of repetitive sequencies belong to the TEs family. Long interspersed element-1 sequences (LINE-1s or L1s) constitute ~17% of the human nuclear DNA, and they contain an internal RNA polymerase II (RNAPII) promoter, two open reading frames (ORF1 and ORF2) and a 3′ UTR containing a polyadenylation signal ending with an oligo(dA)-rich tail of variable length [22]. An estimated 80–100 full-length retrotransposition competent L1s (all belonging to the modern L1 family L1Hs, L1PA1, and L1PA2) are present in a typical diploid human genome. Short interspersed nuclear elements (SINEs) are non-autonomous, non-coding TEs, that are about 100 to 700 base pairs in length. Some SINE sequences, which possess an intact RNA pol III promoter, are functionally active but the majority are not actively transcribed [23]. Alu, belonging to SINE and characteristic of primates, represents one of the most successful of all mobile elements, having a copy number exceeding 1 million copies in the human genome, thus contributing to almost 11% of the human genome. Regarding aging, the expression of several families of TEs appear to be associated with active transposition [2,24,25]. A transcriptional derepression of L1 elements was described during replicative senescence of human fibroblasts [26]. In this condition, the increased L1 expression was associated with the activation of a type I interferon (IFN-I) response [27]. We aimed to verify if L1 methylation status and L1-Alu expression levels are increased in human endothelial cells during replicative senescence, as well as in dermal fibroblasts.

## 2. Materials and Methods

### 2.1. Cell Lines and Cell Culture

Human umbilical vein endothelial cells (HUVECs) and human dermal fibroblasts (NHDFs) were purchased from Clonetics (Lonza, Basel, Switzerland) and cultured in Endothelial Cell Growth Medium (EBM-2, CC-3156, Lonza) and Fibroblast Growth Basal Medium (FBM-2, CC-3131, Lonza) supplemented with endothelial and fibroblast SingleQuot Bullet Kit (CC4176 and CC-3132, Lonza), respectively. The HUVEC and NHDF cells were both seeded and sub-cultured before reaching confluence (70–80%) at a density of 5000/cm^2^ in a humidified atmosphere of 5% CO_2_ at 37°C. All cells tested negative for mycoplasma infection. Population doublings (PDs) were calculated by the formula: (log_10_F − log_10_I)/log_10_2, where F is the number of cells at the end of the passage, and I is the number of seeded cells.

### 2.2. Biomarkers of the HUVEC and NHDF Replicative Senescence

HUVEC and NHDF senescence was characterized by analysing several well-established senescence biomarkers, comparing non senescent (Young) cells, with an intermediate senescence status (Int) and old (Sen) cells. The telomere length was measured using the Cawthon’s method [28]. Telomere/Single copy gene (T/S ratio) of each sample was determined as previously described [29]. For the detection of Senescence-Associated (SA) β-galactosidase activity, HUVECs and NHDFs were cultured until the arrest of replication and classified based on the percentage of SA-β- galactosidase (β-gal) positive cells into young (SA β-gal < 5%), intermediate (40% < SA β-gal < 60%), and senescent (SA β-gal > 80%) using Senescence Detection Kit (cat. no. K320, BioVision Inc., Milpitas, CA, USA).

### 2.3. Genome-Wide DNA Methylation Analysis

Genomic DNA was extracted in triplicate from young and senescent HUVECs using Qiagen’s QiAmp mini kit following the manufacturer’s instructions. Bisulphite-conversion of 1 μg DNA was performed as described in [17]. Briefly, extracted DNA deriving from young and senescent HUVECs, was bisulphite-converted, and analysed by the Infinium Human MethylanEPIC Bead Chip. The CLC Genomics WorkBench software was used to align sequencing data with human genome, promoters, and regulatory regions, while Repeatemasker (rpmsk), downloaded from USCS Genome, served to identify methylations within repeating sequences. Gene density was calculated using the ‘FlankBed’ function of Galaxy, obtaining the sequences 500 Kbase upstream and downstream of each CpG significantly demethylated in senescence cells. Gene densities were divided into three groups: low gene density (1–10 genes), middle gene density (11–40 genes), and high gene density (41–90 genes).

### 2.4. Primer Design

Suitable primers were firstly identified using “Primer 3”. The unique recognition of a single sequence was verified using the function “In silico PCR” of UCSC. Primers sequences were further checked using “Mega-X” and aligning primers sequences with the genome, the positions of demethylated CpGs and the consensus sequences of LINE1, L1PA2 and L1HS. The compatibility of the melting temperature (Tm) of the primers pair and the length of the amplified was also verified.

### 2.5. RNA Extraction

Total RNA, including small (<200 nucleotides) RNAs, was extracted from HUVEC and NHDF pellets using Norgen total RNA Purification Kit (cat. no. 37500, Norgen Biotek Corporation, Canada) according to the manufacturer’s protocol. Purified RNA was stored at − 80 °C until analysis.

### 2.6. Quantitative RT-PCR of Mature microRNAs

The MiRNA expression was measured by Real Time PCR using the TaqMan miRNA assay (cat. no. 4427012 Thermo Fisher Scientific, Waltham, MA, USA) as previously described [30].

### 2.7. mRNA Expression Level

For p16 and p21, 1 ug of purified RNA were retrotranscribed using the PrimeScript™ RT reagent Kit with gDNA Eraser (Perfect Real Time) (TaKaRa), according to the manufacturer’s instructions and assessed as previously described [17]. Primer sequences (written 5′-3′) were listed as follows: p16, Fw: CATAGATGCCGCGGAAGGT, Rv: CTAAGTTTCCCGAGGTTTCTCAGA; p21, Fw:TGGACCTGTCACTGTCTTGT, Rv: TCCTGTGGGCGGATTAG; β-actin, Fw: TGCTATCCCTGTACGCCTCT, Rv: GTGGTGGTGAAGCTGTAGCC and GAPDH, Fw: TGCACCACCAACTGCTTAGC, Rv: GGCATGGACTGTGGTCATGAG. Primer concentration was 200 nM. 2^Δ−Ct^ method was performed to analyse the results and β-actin was used as internal control.

The Alu, ORF2, and L1PA2 expression levels were measured applying the same method described above with some modifications. During the reverse-transcribed reaction, the RT Primer Mix test tube was replaced with oligo (dT). To verify the performance of each experiment, a sample without template (no RT) and a calibrator were run in parallel with the samples. The primers sequences (written 5′-3′) were listed as follows: L1PA2-Chr14, Fw: CAGCTCAGGTCTACAGCTCC, Rv: CCTCGCCTTGCTTCCACTT; Alu, Fw: CGCCTGTAATCCCAGCAC, Rv: TCTCGATCTCCTGACCTCGT; ORFp2, Fw: CAGCCGAATTCTACCAGAGG; Rv: CCGGCTTTGGTATCAGAATG; 5S, Fw: CGATCTCGTCTGATCTC, Rv: CTACAGCACCCGGTATT.

### 2.8. Nucleus/cytoplasm Fractionation and DNA Extraction and Quantification

A sample of 1 × 10^6^ of cultured young and senescent cells were trypsinized and centrifuged to produce a pellet. Nucleus/cytoplasm fractionation was performed as described in [31] with some modifications. Cells were passed 15 times through a 25-gauge needle to enhance cell lysis. The pellet suspension was centrifuged twice at 800 × *g* for 5 min each. The pellet, considered as the nuclear fraction, was resuspended in a lysis buffer (50 mM HEPES, pH 8.0, 150 mM NaCl, 10% glycerol, 1 mM DTT, 2 mM EDTA, 0.5% SDS) and incubated for 1 h at 37 °C while the supernatant, considered as the cytosolic fraction was centrifuged twice at 10,000× *g* for 10 min. The DNA was extracted with QIAamp DNA Blood Mini Kit (QIAGEN, Inc., Valencia, CA, USA) following the manufacturer’s recommendations and treated with 1mg/mL of RNase A, DNase and protease-free (Life Technologies, Carlsbad, CA, USA) for 30 min at 37 °C. The ORF2 and Alu DNA was quantified using RT-PCR using the above-mentioned primers.

### 2.9. Western Blot

The protein concentration was determined using the Bradford method (Sigma–Aldrich, Milan, Italy). A sample of 30 ug of total protein extracts was separated by SDS-PAGE and transferred to nitrocellulose membranes. The membranes were blocked in EveryBlot Blocking Buffer (BioRad, Hercules, CA, USA) for 5 min at room temperature and then incubated overnight at 4 °C with primary antibodies targeting Lamin A/C (Concentration 1:1000, #2032, Cell Signalling Technology, Danvers, MA, USA) and β-Actin (Concentration 1:3000, sc-47778, Santa Cruz Biotechnology, Santa Cruz, CA, USA). After incubation with the specific HRP-conjugated antibody (Vector; 1:10,000 dilution), the chemiluminescent signal was detected using Clarity Western ECL Substrate (BioRad). The autoradiographic films thus obtained were quantified using the Image J software version 1.45 (National Institutes of Health, Bethesda, MD, USA).

### 2.10. Statistical Analysis

Differences in methylation between non senescent and senescent cells were analysed applying Wilcoxon sign ranks, after Bonferroni’s correction. Data are presented as mean ± standard deviation (SD) of at least three independent experiments. The Student’s *t* test was applied to determine differences between samples. The Z-test was used for comparison between proportions. A two-way ANOVA was used to compare expression of retrotransposable elements in the cytosolic and nuclear fractions of HUVEC and NHDF cells. The *p* values < 0.05 were considered significant. Statistical analyses were performed using IBM SPSS Statistics, version 25, and GraphPad Prism 7.

## 3. Results

### 3.1. Characterization of the Senescence Status of HUVECs and NHDFs

The senescent status of HUVECs and NHDFs was characterized by analysing several well-established senescence-associated (SA) biomarkers. Non-senescent (Young), intermediate (Int) and senescent (Sen) cells were analysed.

**Figure 1:**
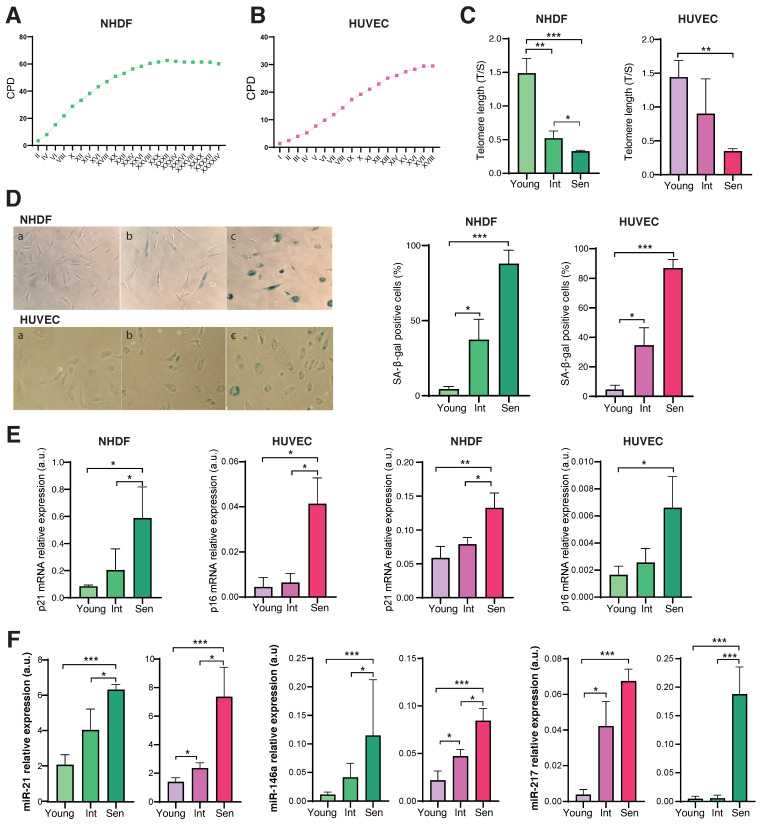
Characterization of replicative senescence of NHDFs and HUVECs. Growth curve showing cumulative population doublings (CPDs) of NHDFs (**A**) and HUVECs (**B**) undergoing replicative senescence (X axis: cell passages). (**C**) Telomere length was analysed by Real Time-PCR calculated as telomere/single copy gene ratio (T/S). (**D**) Representative images of SA- β-Gal in young (a), in an intermediate passage (b) and in senescent (c) NHDFs and HUVECs. (**E**) p21 and p16 mRNA expression evaluated by RT-PCR. β-actin was used as internal control. (**F**) miR-21, miR-146a and miR-217 relative expression evaluated by RT-PCR. RNU44 was used as internal control. * *p* < 0.05; ** *p* < 0.01; *** *p* < 0.001 for paired *t*-test.

Compared with Young cells, Sen cells and both HUVECs and NHDFs, were characterized by growth arrest, which was documented by reduced cumulative population doublings (cPDs) (Figure 1A,B), progressive telomere shortening (Figure 1C), increased SA β-gal activity (Figure 1D), and transcriptional upregulation of the cell cycle regulators p21 and p16(INK4a) (Figure 1E). Finally, the expression levels of innovative biomarkers of cellular senescence, i.e., the inflammamiRs miR-21, miR-146a, and miR-217, that we identified in previous papers [17,32] were analysed in both NHDFs and HUVECs. Significant increased expression of the three microRNAs was observed in Sen vs. Young cells and both NHDF and HUVEC cells (Figure 1F).

### 3.2. The Genome-Wide Methylation Analysis of Young and Senescent HUVECs

The results of the genome-wide methylation analysis carried out on HUVEC cells, comparing young and senescent cells were previously published by our group [17]. Characterization and comparison of the epigenetic profile of old and young cells by Infinium EPIC probes demonstrated a differential methylation state at 335,495 CpG sites. Senescent cells showed significant hypomethylation in “CpG island/shore/shelf regions located in intergenic regions” and significant hypermethylation of “single CpG island/shore/shelf in genes” [17]. A further analysis of the hypo and hypermethylated CpG sites in senescent compared to young HUVECs revealed an increased demethylated CpG in genomic regions characterized by low gene density [17].

To further investigate whether the sequences characterized by increased senescence-associated demethylation belong to transposable elements (TEs), the differentially methylated CpG sites identified by genome-wide methylation analysis were mapped on the reference genome and annotated for their localization inside TE sequences. Only TE elements of the L1 and Alu families were considered for this annotation. The CpGs with significant demethylation (false discovery rate, FDR < 0.05) in senescent HUVECs compared to younger cells were considered in this analysis. The results revealed that most of the CpGs within these two TE families were demethylated in senescent HUVECs compared to younger cells and that most of these sites belong to the LINE1 family, divided in the subfamilies L1M, L1P (evolutionary old LINE1 subfamilies), and L1HS (evolutionary young LINE1 subfamily). Table 1 lists the 15 differentially methylated CpGs within LINE1 and Alu TEs showing the lowest BH-adjusted *p*-values, whereas the complete list of differentially methylated LINE1 and Alu CpGs is available as Supplementary File S1.

The length of each L1 genomic element containing CpG sites demethylated in senescent HUVECs was verified. Almost all these L1s were found to be incomplete, lacking elements essential to their activity, therefore unable to retrotranspose. Importantly, an L1PA2 element (6053 bp long) containing two analysed CpG sites both hypomethylated in old cells (cg19716125 and cg24170212), located on chromosome 14, extending from position 46.352.411 to 46.358.464 on the hg19 human genome reference was almost complete, lacking only few bases at 5′. The L1PA2 are TE sequences not so active as the L1HS, but evolutionarily quite recent; they are chimeric and present the 5′ promoter sequence typical of LINE1 [33]. The characteristics of this L1PA2, demethylated in senescent HUVECs and quite complete in sequence, suggest that it could be able to transpose.

**Figure 2:**
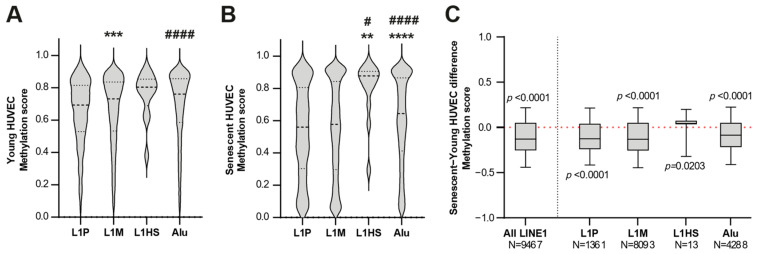
Methylation score of LINE subfamilies and Alu. (**A**,**B**) Violin plots showing the methylation score of the three LINE subfamilies and Alu in (**A**) young and (**B**) senescent HUVECs. ** *p* < 0.01; *** *p* < 0.001; **** *p* < 0.0001 vs. L1P. # *p* < 0.05; #### *p* < 0.0001 vs. L1M for two-tailed unpaired *t*-test. (**C**) Boxplots showing the distribution of differences in the methylation scores of the LINE1 family, subfamilies, and Alu between senescent and young HUVECs. *p* for Wilcoxon signed-rank tests.

This analysis was performed including all CpG genomic sites identified by InfiniumEPIC probes and mapped within the three analysed L1 subfamilies (L1M, L1P and L1HS). Figure 2A,B shows the methylation scores within each LINE1 subfamily in young and senescent HUVECs, respectively, while the boxplots in Figure 2C summarize the distributions of senescent vs. young methylation differences (beta-values). Notably, an enrichment of demethylated sites was observed for the LINE1 family (*p* < 0.001), in particular, in the L1P and L1M subfamilies, which include >99.8% of the differentially methylated sites. On the contrary, an hypermethylation pattern was observed in the remaining CpGs within the L1HS subfamily.

As the methylation status of TEs could be affected by their genomic context, including the proximity to functional elements [25,34,35] the genomic distribution of the significantly demethylated LINE1 CpGs was compared across regions of different gene density. In particular, the gene density in 1 Mb regions centred on the position of each CpG was considered and expressed as classes of 10 genes/Mb (e.g., class 1 = 0–10 genes/Mb, class 2 = 11–20 genes/Mb, […], class 17 ≥ 160 genes/Mb).

**Figure 3:**
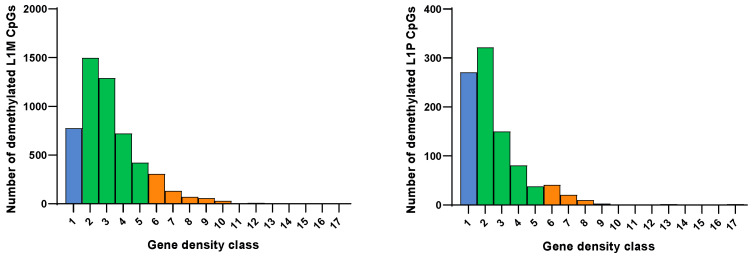
Distribution of significantly demethylated CpGs within LINE1 subfamilies L1M and L1P grouped according to gene density classes. Low gene density (class 1), blue; intermediate gene density (classes 2–5); green; high gene density (classes 6–17), orange.

Figure 3 shows the frequencies of demethylated CpGs within L1M (total *n* = 5347) and L1P (total *n* = 943). The CpGs within the L1HS subfamily were not further considered, given their low abundance (*n* = 1). Demethylated L1M and L1P elements can be mapped both in areas with low and with high gene density.

More in detail, we observed an enrichment of demethylated LINE1 CpGs surrounded by regions with low gene density (range from 1–10 genes per Mb) compared to the whole population of LINE1 CpGs (*p* < 0.00001) (Table 2). This was particularly evident for CpGs belonging to the L1M subfamily, while the enrichment failed to reach statistical significance for the L1P subfamily.

Because recent data suggest that cellular senescence is characterized by a strong activation of the TEs [27,36], we extended our analysis to the model of replicative senescent dermal fibroblasts (NHDF).

### 3.3. Cellular Expression and Compartmentation of Alu and LINE1 Transcripts

**Figure 4:**
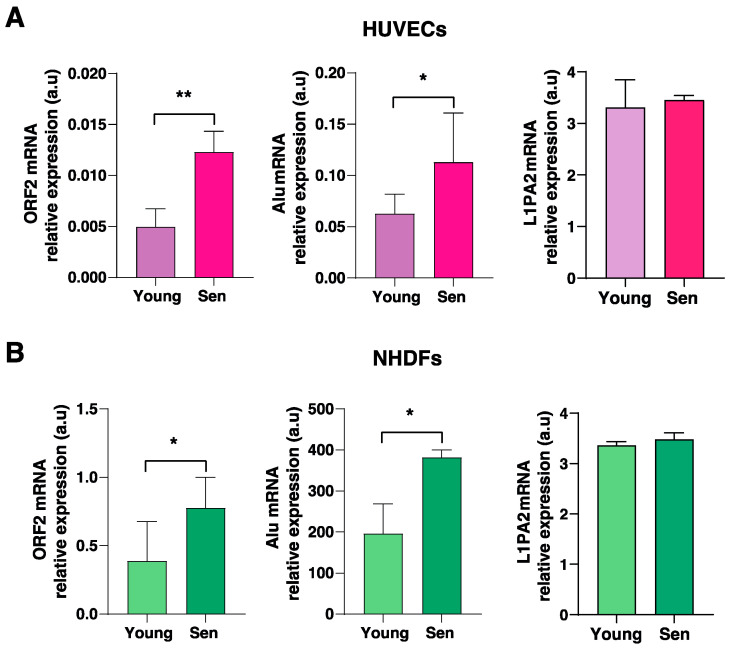
Relative expression of ORF2, Alu retrotransposable elements, and L1PA2 in (**A**) HUVEC and (**B**) NHDF cells. * *p* < 0.05; ** *p* < 0.01 for two-tailed paired *t*-test.

To verify the hypothesis that L1 demethylation in senescent cells could be associated with L1 transcription/activation, we analysed the mRNA expression levels of the Alu family of Short Interspersed Nuclear Elements (SINEs), dependent on the ORF2 enzyme—encoded by LINE sequences—for its reverse transcription and retrotransposition. We selected Alu elements because increased evidence has suggested an association of this SINE family with cellular senescence process in fibroblasts [27]. The analysis of RNA transcripts revealed significant increased expression of both Alu elements and LINE1, in senescent HUVECs (Figure 4A) and NHDFs (Figure 4B) compared to the younger ones.

We also assessed the levels of L1PA2, which was identified by the previous analysis as the most demethylated full-length LINE1 element in senescent cells, suggesting that it could be potentially active. Notably, L1PA2 RNA was detectable both in senescent and young cells, suggesting that this LINE sequence is effectively transcribed both in endothelial cells and fibroblasts; however, no significant modulation of its expression was found in senescent cells (Figure 4).

Then, to clarify whether the cytoplasm of senescent cells is enriched in retrotranscribed TE sequences, we analysed the abundance of the selected TEs in both the nuclear and cytosolic fractions. An increase in TEs DNA copies in the nucleus would be suggestive of an enhanced sequence integration, whereas an increased number of TEs DNA copies in the cytoplasm could indicate an increase in the retrotranscription process, which proceeds through a DNA intermediate. The correct separation of nuclear and cytoplasmatic fractions was confirmed by analysing the expression levels of the cytoplasmatic protein β-actin and of the nuclear protein Lamin A/C. Western Blot analysis confirmed the absence of cross-contaminations between nuclear and cytoplasmatic fractions (Figure S1).

**Figure 5:**
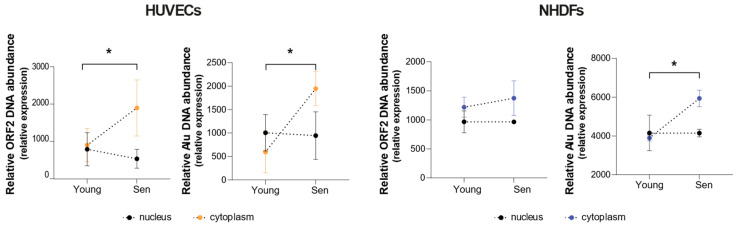
DNA quantification of ORF2 and Alu retrotransposable elements in the cytosolic and nuclear fractions of HUVEC and NHDF cells. * *p* < 0.05 for two-way ANOVA.

A significantly increased abundance of DNA sequences deriving from Alu was observed in the cytoplasmic fraction of senescent HUVECs and NHDFs, whereas an increased abundance of sequences derived from ORF2 was observed only in the cytoplasm of senescent HUVECs (Figure 5).

Regarding the nuclear fraction, no significant senescence-associated differences in the levels of Alu and ORF2 were observed.

## 4. Discussion

Here, we analysed some epigenetic mechanisms that could be involved in the acquisition of the senescent phenotype in two well established replicative senescence cellular models, human endothelial cells (HUVECs) and fibroblasts (NHDFs). Our previous published results of genome-wide methylation analysis performed in young and senescent HUVECs, revealed a global genome demethylation in senescent HUVEC cells [17]. Starting from these results we aimed to clarify if the most significantly demethylated single CpG sites, identified in this genome-wide methylation analysis, could belong to TE sequences. To achieve this aim, the most significantly demethylated single CpG sites were mapped on the reference genome and annotated for their localization inside TE sequences, revealing a significant senescence-related hypomethylation of sequences belonging to the TE L1 family, divided in the three subfamilies L1M, L1P, and L1Hs. Interestingly, significant increased demethylated CpG loci were identified in genomic regions with low coding gene density.

This first part of the analysis suggests that cells can adopt different mechanisms of self defence against TEs, such as the methylation and localization of potentially more active TE sequences in regions with a reduced number of coding genes. Increasing evidence suggested that L1 methylation status can be related to the development of some Aging-related disorders (ARDs) or related risk factors, including obesity [37], dyslipidemia [38], type 2 diabetes mellitus (T2DM) [39], cancer [40,41], and neurodegenerative diseases (reviewed in [42]). As almost all the most common ARDs share some pathological features, including endothelial dysfunction [43], the main finding of our research was the analysis of human endothelial cellular models to pave the way for ex vivo and/or in vivo studies in patients affected by ARDs.

We further verified the integrity and completeness of the TEs containing significantly demethylated CpGs in senescent cells. Almost all the TEs were found to be incomplete and, therefore, unable to retrotranspose. Importantly, only one L1PA2 element (6053 bp long), located on chromosome 14, was almost complete. Full-length L1 transcription is driven by a CpG dinucleotide-rich internal promoter, and an hypomethylation of L1 can cause their activation with consequent retroelement transposition and chromosomal alteration [44]. Therefore, we further verified the expression levels of the L1 elements, the specific (hypomethylated) L1PA2 element in chromosome 14, and Alu belonging to SINEs. In the human genome, the L1 and Alu elements are the two most abundant families of TEs. Part of their high prevalence may be explained by the facts that L1s appear to be the only currently active autonomous TEs in the human genome, and Alu elements can hijack the L1 machinery, especially L1 ORF2, for the retrotranscription and transposition [45]. These analyses were performed both in HUVEC and in NHDF cells. The analysis of the TE RNA expression revealed a significant increase of both Alu and L1 RNA transcripts in senescent HUVECs and NHDFs. The increased RNA copies of Alu and L1 in senescent compared to younger cells, suggest a senescence-associated activation of these TEs. Our data are in accordance with recent evidence on artificially induced senescent cell-lines and senescent human hematopoietic stem and progenitor cells (HSPCs), confirming a robust activation of TEs [46,47]. Our results are in accordance also with previous studies, showing that L1 increased expression was associated to a senescence-like state in cancer cell lines [48], normal human fibroblasts, and adult mesenchymal stromal cells [49].

Notably, L1PA2 RNA was not significantly increased in senescent cells, suggesting that even if L1PA2 is demethylated in senescent HUVECs, it is not actively transcribed. As the relationship between CpG methylation and gene expression is not always predictable, testing of a wide range of transcripts would be required to draw definitive conclusions on the senescence-related upregulation of TEs. Here, by showing that the RNA of the L1-encoded polypeptide ORF2 is upregulated in senescent cells, and that an increased abundance of cDNA sequences deriving from ORF2 retrotranscription could be retrieved in the cytoplasm of these cells, we have provided evidence supporting the notion that the general hypomethylation of TEs could be associated with the activation of a machinery leading to the accrual of cytoplasmic nucleic acids.

Increased evidence suggests that cytoplasmic nucleic acids can activate the cytoplasmic sensor of nucleic acids, thus activating an antiviral response promoting type 1 IFN increased expression [50]. More in detail, the accumulation of TEs in the cytoplasm could trigger the cGAS-STING-IRF3 axis activation, thus inducing an increased transcription of type 1 IFN genes [51].

An important limitation of the present study needs to be acknowledged. The LINE-1 and Alu analysed in the bead arrays were an extremely small fraction of the L1 and Alu total elements in the human genome. In addition, for each of these sequences, only one or a few CpG were analysed; consequently, the overall conclusions of our research are new and of potential interest in human ARDs, but not necessarily related to all the L1 and Alu families interspersed in the human genome.

Overall, our results suggest that the demethylation of specific regions in the genome of senescent cells, could contribute to activate TEs, that can be transcribed as RNA not only in fibroblasts but also in endothelial cells. This process was linked to increased DNA damage, altered regulation of the host genome, and cytoplasmic accumulation of retrotransposon-derived cDNAs that can promote sterile inflammation, thus contributing to the deleterious effects of aging [52].

Our data suggest a complex crosstalk between different epigenetic mechanisms in cellular senescence, converging on the promotion and maintenance of a proinflammatory secretome that can spread proinflammatory molecules at paracrine and systemic levels, thus fuelling inflammaging and increasing the chances to develop the most common ARDs.

## Figures and Tables

**Table 1:** Methylation differences of TEs between senescent and young HUVECs. Methylation of CpG sites is expressed as DNAm score, ranging from 0 to 1. ↑ indicates hypermethylation of the specific CpG in senescent HUVECs compared to young HUVECs. ↓ indicates demethylation of the specific CpG probe in senescent HUVECs compared to young cells.

Chromosomal Location	CpG IDs	Repeat Masker Annotation	DNAm Young HUVEC	DNAm Senescent HUVEC	DNAm Difference (Senescent − Young)	*p*-Value	Adjusted BH *p*-Value
Chr3: complement (127793383-4)	cg13228884	L1ME2z	0.703	0.045	0.658 ↓	1.85 × 10^−8^	1.04 × 10^−4^
Chr4: complement 84255411-2	cg25428494	L1PB4	0.693	0.077	0.616 ↓	1.91 × 10^−8^	1.01 × 10^−4^
Chr5: complement (180561011-2)	cg15102731	L1MEg	0.879	0.092	0.787 ↓	4.13 × 10^−9^	8.82 × 10^−5^
Chr7: (80642464-5)	cg17270475	L1MEg1	0.192	0.936	0.744 ↑	3.52 × 10^−8^	1.17 × 10^−4^
Chr7: complement (157706117-8)	cg13117953	L1ME3E	0.605	0.039	0.566 ↓	8.52 × 10^−10^	6.65 × 10^−5^
Chr13: complement (113707783-4)	cg00245856	L1MC4	0.862	0.107	0.755 ↓	4.89 × 10^−8^	1.25 × 10^−4^
Chr14: complement (46358139-40)	cg24170212	L1PA2	0.891	0.422	0.469 ↓	1.19 × 10^−8^	9.97 × 10^−5^
Chr15: complement (22562214-5)	cg07397108	L1MEf	0.636	0.152	0.484 ↓	4.41 × 10^−8^	1.21 × 10^−4^
Chr17: complement (33780343-4)	cg06138714	L1ME1	0.736	0.036	0.700 ↓	1.15 × 10^−8^	9.97 × 10^−5^
Chr17: complement (45946982-3)	cg06085038	L1ME3A	0.823	0.616	0.207 ↓	1.15 × 10^−8^	9.97 × 10^−5^
Chr20: complement (58106111-2)	cg19748181	L1MC1	0.889	0.109	0.780 ↓	4.14 × 10^−8^	1.20 × 10^−4^
Chr21: complement (19195209-10)	cg25387466	L1MEc	0.729	0.121	0.608 ↓	1.10 × 10^−8^	9.97 × 10^−5^
Chr1: complement (158904784-5)	cg25464787	AluSq2	0.822	0.088	0.734 ↓	9.41 × 10^−9^	9.97 × 10^−5^
Chr7: complement (1253999-4000)	cg15877936	AluSg	0.767	0.195	0.572 ↓	2.97 × 10^−8^	1.14 × 10^−4^
Chr16: complement (8974513-4)	cg06611212	AluSp	0.810	0.588	0.222 ↓	4.42 × 10^−8^	1.21 × 10^−4^

DNAm, DNA methylation score; BH, Benjamini–Hochberg.

**Table 2:** LINE1 subfamily distributions of demethylated CpGs, respectively in areas of low (0–10 genes/Mb), intermediate (11–50 genes/Mb), and high gene density (>50 genes/Mb).

	Low Gene Density	Intermediate Gene Density	High Gene Density	Total	Z-Test (Low Gene Density)
Demethylated LINE 1 CpGs	1046 (16.6%)	4528 (72.0%)	717 (11.4%)	6291 (100%)	*p* < 0.00001
All LINE1 CpGs	3457 (12.1%)	22,076 (77.5%)	2940 (10.3%)	28,473 (100%)	Ref.
Demethylated L1M CpGs	775 (14.5%)	3936 (73.6%)	636 (11.9%)	5347 (100%)	*p* < 0.00001
All L1M CpGs	2330 (9.6%)	19,246 (79.4%)	2668 (11.0%)	24,244 (100%)	Ref.
Demethylated L1P CpGs	271 (28.7%)	591 (62.7%)	81 (8.6%)	943 (100%)	*p* = 0.136
All L1P CpGs	1099 (26.4%)	2798 (67.1%)	272 (6.5%)	4169 (100%)	Ref.

Results of Z-tests testing the hypothesis that the proportion of demethylated LINE1 CpGs in areas of low gene density is higher than expected (Ref.) are reported.

## Data Availability

The data presented in this study are available on request from the corresponding author.

## Supplementary Materials

The following supporting information can be downloaded at https://www.mdpi.com/article/10.3390/cells11233799/s1. Figure S1: Western blot analysis of Lamin A/C and B-actin in cytoplasm (C) and nuclear (N) fraction of young and senescent HUVECs and NHDFs. Supplementary File S1. Methylation differences of LINE1/Alu transposable elements between senescent and young HUVECs. Methylation of CpG sites is expressed as a DNAm score, ranging from 0 to 1. CpG probes with a Benjamini–Hochberg adjusted *p*-value < 0.05 are displayed.
