# Supplementary material for: Replicative Senescence-Associated LINE1 Methylation and LINE1-Alu Expression Levels in Human Endothelial Cells
Source: Cells. 2022 Nov 27;11(23):3799. doi: 10.3390/cells11233799 (PMC9739197; doi:10.3390/cells11233799)

Figure S1. Western blot analysis of Lamin A/C and B-actin in cytoplasm (C) and nuclear (N) fraction of young and senescent HUVECs and NHDFs.

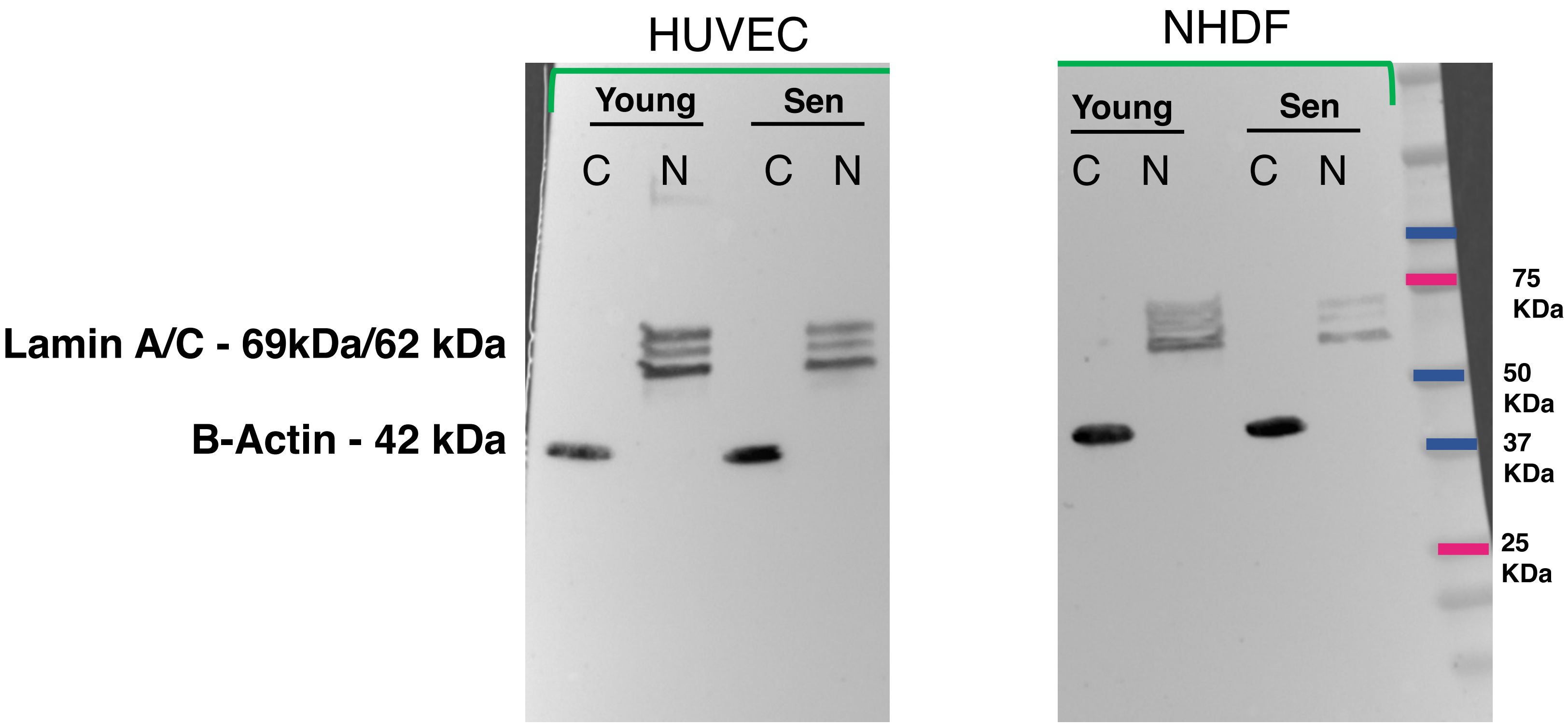

Supplement: Supplementary file 1 [file cells-11-03799-s001.zip › Figure S1.pdf]
